# A novel autoantibody test for the detection of pre-neoplastic lung lesions

**DOI:** 10.1186/1476-4598-13-78

**Published:** 2014-04-05

**Authors:** Frazer J Lowe, Weike Shen, Jinchi Zu, Junfu Li, Hao Wang, Xufei Zhang, Li Zhong

**Affiliations:** 1 British American Tobacco (Investments) Ltd, Group Research and Development, Regents Park Road, Millbrook, Southampton SO15 8TL, UK; 2 Hebei University, 180 Wusi Rd., Baoding, China; 3 Western University of Health Sciences, 309 E. 2nd St, Pomona, CA, USA

**Keywords:** Lung cancer, Smoking, Biomarker, Atypical adenomatous hyperplasia, Squamous cell dysplasia, Autoantibody

## Abstract

**Background:**

Atypical adenomatous hyperplasia (AAH) and squamous cell dysplasia (SCD) are associated with the development of malignant lesions in the lung. Accurate diagnosis of AAH and SCD could facilitate earlier clinical intervention and provide useful information for assessing lung cancer risk in human populations. Detection of AAH and SCD has been achieved by imaging and bronchoscopy clinically, but sensitivity and specificity remain less than satisfactory. We utilized the ability of the immune system to identify lesion specific proteins for detection of AAH and SCD.

**Methods:**

AAH and SCD tissue was surgically removed from six patients of Chinese descent (3 AAH and 3 SCD) with corresponding serum samples. Total RNA was extracted from the tissues and a cDNA library was generated and incorporated into a T7 bacteriophage vector. Following enrichment to remove "normal" reactive phages, a total of 200 AAH related and 200 SCD related phage clones were chosen for statistical classifier development and incorporation into a microarray. Microarray slides were tested with an independent double-blinded population consisting of 100 AAH subjects, 100 SCD subjects and 200 healthy control subjects.

**Results:**

Sensitivity of 82% and specificity of 70% were achieved in the detection of AAH using a combination of 9 autoantibody biomarkers. Likewise, 86% sensitivity and 78% specificity were achieved in the detection of SCD using a combination of 13 SCD-associated markers. Sequencing analysis identified that most of these 22 autoantibody biomarkers had known malignant associations.

**Conclusions:**

Both diagnostic values showed promising sensitivity and specificity in detection of pre-neoplastic lung lesions. Hence, this technology could be a useful non-invasive tool to assess lung cancer risk in human populations.

## Background

Carcinoma of the lung is the leading cause of morbidity and mortality of human solid cancer worldwide. It accounted for around 12.7% of all new cancer incidences and 18.2% of all cancer mortality, or approximately 1.4 million deaths worldwide in 2008 [1]. In populations with long-term cigarette use, the proportion of lung cancer cases attributable to smoking approaches 90% [2]. In Asia, particularly in China, rising smoking rates cause the incidence of lung cancer to continue to increase. Despite advances in therapy, early detection of lung carcinoma is critical to facilitate successful treatment and increase the chances of survival. Five year survival rates for non-small cell lung cancer diagnosed between the years of 1990 and 2001 were reported to be 61% for stage 1A, compared to 34%, 13% and 1% for stages IIA, IIIA and IV respectively [3]. Thus, novel techniques which facilitate accurate diagnosis of early malignancy or pre-malignancy would be of great benefit to improve survival rates.

Atypical adenomatous hyperplasia (AAH) and squamous cell dysplasia (SCD) have been reported as precursors of adenocarcinoma and squamous cell carcinoma of the lung respectively [4,5]. The progression of healthy tissue to pre-neoplastic lesions and malignant disease has been described previously by Wistuba and Gazdar [6]. Briefly, with respect to squamous cell carcinoma, loss of heterozygosity (LOH) on chromosomes 3p21 and 9p21 can lead to telomerase dysfunction. DNA methylation in cell cycle regulatory genes such as p16INK4a, and further LOH in tumor suppressor genes such as FHIT and TP53 contribute further to the dysregulation of cell growth in the epithelial tissue, ultimately leading to carcinoma *in situ* and metastatic disease. With respect to adenocarcinoma, mutations in the *ras* oncogene pathway (smokers) or the epidermal growth factor receptor pathway (non-smokers) can lead to tissue growth dysregulation, and ultimately adenocarcinoma.

Currently, the most widely used clinical methods for the detection of pre-neoplastic lung SCD lesions include white light bronchoscopy (WLB) and auto-fluorescence bronchoscopy (AFB). Chen et al. [7] reviewed 492 publications and 14 studies contained data which was suitable to conduct a meta-analysis (15 data sets) in which WLB and AFB were compared for sensitivity and specificity (results were confirmed by tissue histology). The pooled sensitivity and specificity of AFB was 0.90 (95% CI 0.84-0.93) and 0.56 (95% CI 0.45-0.66) compared to 0.66 (95% CI 0.58-0.73) and 0.69 (95% CI 0.57-0.79) for WLB. Thus, AFB was reported to be superior to WLB for the detection of lung cancer and pre-neoplastic SCD lesions. The technique does however have some limitations with respect to deployment in large clinical studies and population studies. AFB requires specialized equipment and trained operators to carry out the procedure and interpret the data. In addition, the procedure itself involves the insertion of an endoscope into the lungs of individuals, and hence the procedure is fairly invasive relative to a blood sample.

AAH arises in the peripheral lung parenchyma where is mainly involved in the gas exchange at the alveolar level. Lesions in AAH are usually small, asymptomatic and radiologically invisible, helical (Spiral) CT combined with surgical biopsy is the most commonly reported method for diagnosis of AAH. At an almost indistinguishable point, AAH lesions can become bronchoalveolar cell carcinoma (BAC) [8]. Clinically it has been suggested using size of the lesion to distinguish between AAH (size ≤ 5 mm) and BAC/Adenocarcinoma (> 5 mm) [8]. Despite some successes in the screening of high risk individuals in terms of lung cancer detection, difficulties still exist with the differentiation of bronchioalveolar carcinoma, adenocarcinoma and AAH with Helical CT alone and false-positive rates for malignancy diagnoses are 20-35% [9,10].

Departing from these more invasive detection procedures, using blood-borne biomarkers for early cancer detection has generated great interest. Detection of serum antibodies against tumor-associated antigens (TAAs) may provide more reliable information for early cancer diagnosis [11,12]. The immune system is sensitive enough in detecting very low levels of TAAs that may originate in only a few neoplastic cells by generating very high affinity T cells and antibodies [13]. Autoantibodies to p53 have been reported in patients with early stage ovarian or colorectal cancers [13], and a panel of serum antibodies can detect non-small cell lung cancer (NSCLC) 5 years prior to autoradiograph detection [14]. Thirty percent of patients with ductal carcinoma in situ in which the proto-oncogene HER-2/neu is overexpressed have serum antibodies specific to this protein [15]. Therefore, it is logical and practical to employ the body’s endogenous immune system as a natural "amplification strategy" to detect the pre-neoplastic lesions of the lung.

The current study utilizes lesion-specific autoantibodies present in blood samples to detect the presence of AAH and SCD lung lesions. This approach could be a useful non-invasive tool to assess lung cancer risk in human populations.

## Results

### Biopanning the phage libraries

Two T7 cDNA phage libraries were constructed using pooled AAH or SCD tissues. The quality of the libraries was titered by plaque assay and found to contain 5.5 × 10^6^ primary recombinants for the AAH library and 3.8 × 10^6^ primary recombinants for the SCD phage library. A PCR test was then run to determine the sizes of individual phage inserts using T7 primer and the results showed the ranges of the cDNA inserts from both the libraries were from 0.5 ~ 2 Kb, indicating both were good phage libraries.

**Figure 1:**
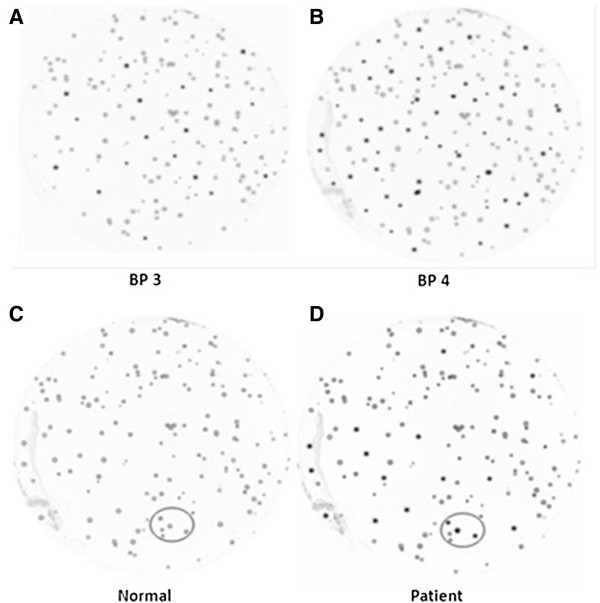
**Biopanning enrichment of immunogenic tumor-associated proteins.** To confirm the enrichment of the biopanning, the outputs of biopans 1–4 (BP1-BP4) were plated onto LB-Agar plates in limiting dilution and plaque lifts were performed. **(A)**. the output of BP3 revealed an increasing number of immunoreactive phage clones than **(B)**. BP4, and illustrates the ability of the sequential biopans to enrich the concentration of tumor-associated proteins recognized by antibodies in patient serum. To confirm the specificity of enriched phage proteins, two nitrocellulose members were lifted twice on the same LB-Agar plate of BP4. One membrane was probed with pooled AAH/SCD patient sera **(D)** while the other was probed with pooled normal sera **(C)**. Numerous immunoreactive clones show on the membrane incubated with patient sera **(D)** that are not seen in the identical membrane incubated with normal serum **(C)**.

The two libraries were then pooled together and biopanned using pooled AAH/SCD and normal serum samples to screen potential tumor-associated antigens expressed by the T7 cDNA phage libraries. Using patient sera as a source of primary antibody, immunodetection revealed that repeated cycles of panning and phage amplification yields an enriched population of immunoreactive phages (Figure 1). Comparison between duplicate plaque lifts of biopan 4, incubated with pooled patient sera or normal sera used in the biopan, showed the ability of this process to select immunogenic phage-expressed proteins (Figure 1). Immunoreactivity of multiple clones with patient sera indicated high affinity binding of these phage-expressed proteins with antibodies in patient sera. In contrast, these same clones are not immuno-reactive in the identical membrane incubated with normal serum. The background seen on the membrane incubated with the normal sera was considered nonspecific reactivity with phage proteins.

### High-throughput screening

**Figure 2:**
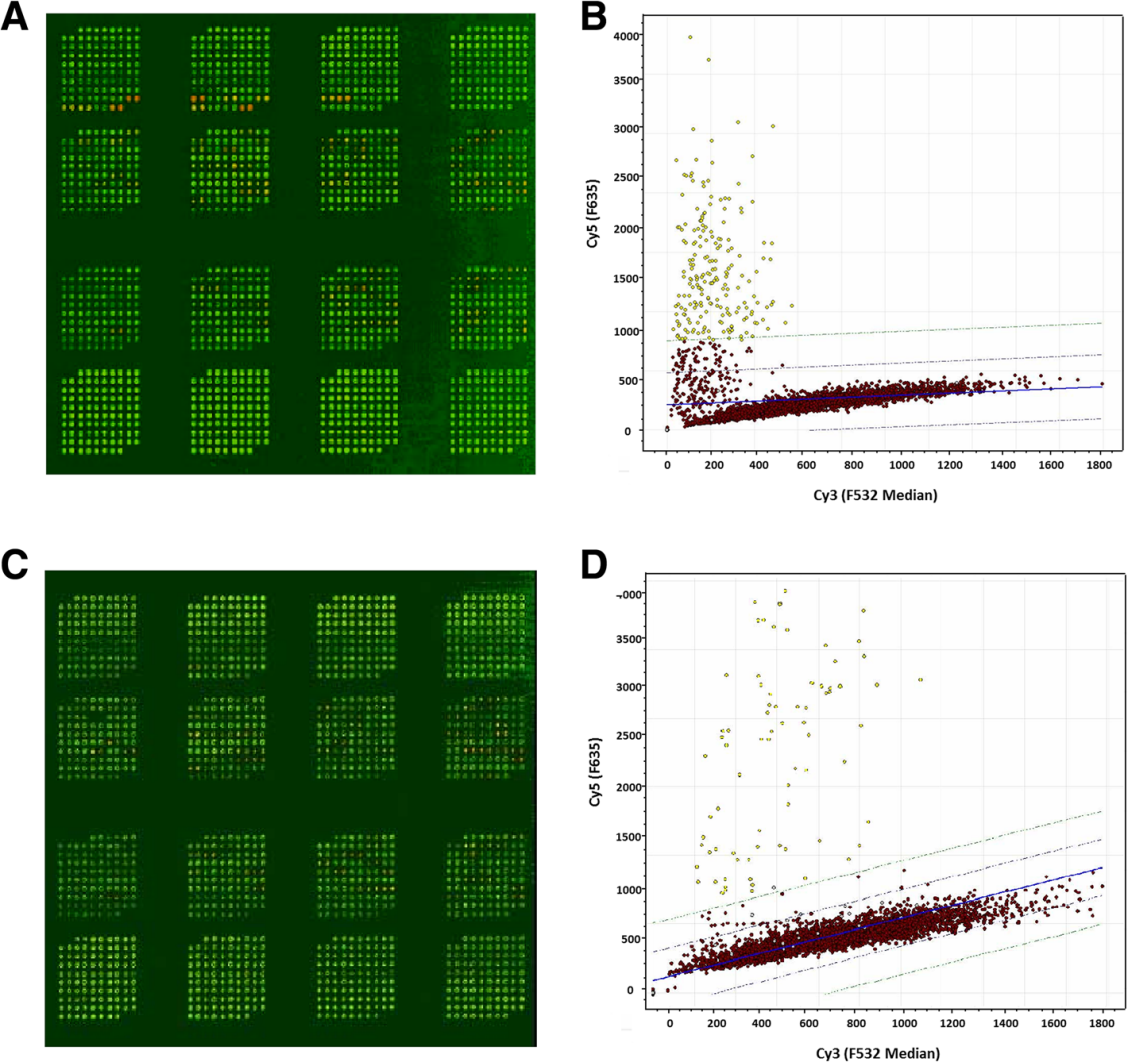
**High-throughput screening of tumor-associated phage proteins.** Biopanned phage clones were spotted on microarray slides and tested with patient serum samples. The partial array images show reactivity patterns for **(A)** AAH and **(C)** SCD patient samples, and the corresponding scatter plots show possible disease-associated phage clones (X-axis is Cy3 signal, Y-axis is Cy5 signal) in **(B)** and **(D)** respectively. The computer-generated regression line and standard deviation lines on the scatter plots assist in identifying candidate marker phages (yellow dots) for diagnostic chip construction.

A total of 4000 phage clones were randomly selected from the output of the BP4 phage library and were spotted on membrane coated microarray slides. These phage clones were then screened with 5 individual AAH or 5 individual SCD patient serum samples that were not used in the biopan to identify disease-associated immunogenic phages. Linear regression of the Cy5:Cy3 signal revealed that 238 individual phages from AAH samples screening and 193 individual phages from SCD screening had signal ratios greater than 2 standard deviations from the average and were chosen as candidates for a "diagnostic chip" construction. An example of linear Cy5:Cy3 regression used for phage selection from a screening chip is shown in Figure 2.

### Statistical analysis in training test

Four hundred immunoreactive phages identified by high-throughput screening (described above), plus 200 "empty" T7 phages, were combined, re-amplified, and spotted in duplicate onto membrane coated slides (Schott, Germany) as diagnostic chips. Replicate chips were used to assess 50 AAH and 50 SCD along with 100 control serum samples as a training test. Normalized Cy5:Cy3 ratios for each 400 phage clones were independently analyzed for statistical significance between patient and normal samples. A Student *t* test was run with a statistical cut-off (*P* < 0.01) that suggested the relative predictive value of each candidate marker. Of the 400 candidate phage proteins, 47 in AAH and 39 in SCD test met the cut-off (p < 0.01).

Based on the *P* values, the top 30 phage clones were collected, combined and then analyzed using a logistic regression algorithm for the most optimal sensitivity and specificity in distinguishing patients and normals. The most predictive accuracy was achieved in AAH test with sensitivity of 92.3% and specificity of 90.2% using 9 combined markers. These results were further validated using leave-one-out cross-validation (LOOCV) between patient and control samples, and ROC curves were generated and yielded an AUC = 0.874. Therefore, combination of 9 phage proteins showed the most accurate and stable classifier in prediction of AAH samples. The same analysis was used for the SCD test, and the result showed that the most optimal sensitivity and specificity in distinguishing SCD from control samples was 98.3% and 95.6% respectively. These results were further validated using leave-one-out cross-validation (LOOCV) between patient and control samples, and ROC curves based on logistic regression were generated and yielded an AUC = 0.959. Therefore, a combination of 13 phage proteins was the most accurate and stable classifier in prediction of SCD samples.

### Independent validation test

**Figure 3:**
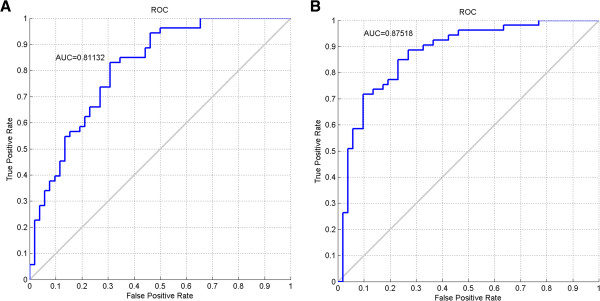
**Diagnostic accuracy for predicting the pre-neoplastic samples in validation test.** Classifiers generated in the training tests were used to predict the samples statures in the blinded validation test. Corresponding receiver operating characteristics (ROC) curves and the value of the area under the curve (AUC) were calculated. Panel **A** was generated in the validation test based on the values from 100 AAH samples and 100 control samples. Panel **B** was generated based on the values from 100 SCD samples and 100 control samples.

Using the same "diagnostic chips" developed in the training test, an independent cohort of 400 serum samples consisting of 200 controls, 100 AAH and 100 SCD serum samples were assayed. The samples were tested in a blinded fashion, and each sample’s status was calculated separately using the AAH or SCD classifier. The final results were checked with the true statuses of the samples, and the sensitivities and specificities were then calculated. Overall we have correctly predicted 168 patients (AAH and SCD) out of 200 disease samples (the sensitivity is 84%), and correctly predicted 156 out of 200 control samples (the specificity is 78%) (Table 1). Corresponding ROC curves and the value of the area under the curve (AUC) were calculated with AUC = 0.81 and AUC = 0.88 for AAH and SCD detection, respectively (Figure 3). As shown in Table 1, the two classifiers have overlaps in predicting AAH and SCD samples even though the sensitivities were rather different. In the other words, the same sample could be identified as either AAH or SCD by the two classifiers. Therefore, further confirmation may be needed in conjunction with radio-imaging or bronchoscopy in clinic.

**Table 1:** Diagnostic accuracies using the two classifiers in the validation test

	**Control samples**	**AAH samples**	**SCD samples**
	**(n = 200)**	**(n = 100)**	**(n = 100)**
AAH classifier prediction rate	140/200	82/100	61/100
Sensitivity		82%	61%
Specificity	70%		
SCD classifier prediction rate	156/200	66/100	86/100
Sensitivity		66%	86%
Specificity	78%		

### Detection of early malignancy in the lung

The World Health Organization has defined the SCD as a pre-neoplastic lesion for squamous cell carcinoma (SCC) [2], which is one step earlier than carcinoma *in situ* (CIS, stage 0 of SCC). Likewise, the AAH is the progenitor lesion for adenocarcinoma, which may progress further to become a bronchoalveolar cell carcinoma (BAC, stage 0 of adenocarcinoma) [6]. In many cases, SCD and CIS, and AAH and BAC are coexistent. Therefore, by separately analyzing the two pairs we may test the ability of the classifiers in detecting the stage 0 of non-small cell lung cancer (NSCLC). According to our samples pathology reports, there were 39 AAH samples that also had BAC, and 42 SCD samples that coexisted with CIS. Using the AAH or SCD classifiers, 30/39 (sensitivity of 76.9%) of AAH/BAC samples were identified by AAH classifier, while 37/42 (sensitivity of 88.1%) of SCD/CIS samples were identified by SCD classifier (Table 2). The AAH classifier showed a slight decrease in detecting AAH/BAC samples, while SCD classifier showed a slight increase in detection of SCD/CIS samples. In order to increase the detection accuracy for the early stage NSCLC, it may need to incorporate some NSCLC specific biomarkers into the present classifiers.

**Table 2:** The accuracies of the classifiers in detection of stage 0 NSCLC

	**AAH samples**	**AAH/BAC samples**	**SCD samples**	**SCD/CIS samples**
	**(n = 61)**	**(n = 39)**	**(n = 58)**	**(n = 42)**
AAH classifier	52/61	30/39		
SCD classifier			49/58	37/42
Sensitivity	85.2%	76.9%	84.5%	88.1%

### Sequence analysis of phage-expressed proteins

The 22 phages (9 for AAH and 13 for SCD) that were chosen for either AAH or SCD classifier development were sequenced. Although the identities of the phage-expressed proteins were not critical for use in a diagnostic assay, the sequences were compared with the GenBank database to determine possible identities. Following sequencing analysis, some proteins are known to be associated with cancer while others have no known association with cancer. Detailed identities are listed in Table 3. Interestingly, there were 3 identical proteins that were selected for both the classifier constructions, which may explain the overlaps for prediction of the disease status. Since these 3 markers weighted heavily in the logistic regression model, the decision was taken to keep these proteins in the array.

**Table 3:** Identities of 9 selected phage proteins for AAH classifier construction and 13 phage proteins for SCD classifier construction

**AAH**				
LTBP1*^,^	BMI1*^,^	GAGE7*^,^	AGBL5	HES1*
PDE4A*	NEFH*	HSPA8*	cDNA FLJ45990	
**SCD**				
LTBP1*^,#^	BMI1*^,#^	GAGE7*^,#^	NRP1*	ADPGK
PRDX4*	KDELR1	SEC61β*	FZD6TFIP11	LGR6*
TXNL2*	KLK2*	BIRC3*		

*Proteins with known malignant association. ^#^Proteins are also present in AAH classifier.

## Discussion

Some lung cancers, such as squamous cell carcinoma and adenocarcinoma appear to develop over years or decades via a series of progressive morphological changes with correlating molecular alterations [6]. The 2004 WHO classification recognises several pre-neoplastic precursor lesions for lung cancer [2]. These include SCD and AAH for squamous cell carcinoma and adenocarcinoma respectively. These aberrant alterations can be detected by the immune system with corresponding autoantibodies production [13]. In this study, a blood-based autoantibody test was successfully developed using phage-display and protein microarray techniques. The current autoantibody chip achieved a sensitivity and specificity of 82% and 70% for AAH, and 86% and 78% for SCD detection, respectively. The respective pooled sensitivity and specificity in a meta-analysis of 14 studies by Chen et al. [7] was 90% and 56% for AFB and 66% and 69% for WLB, respectively, in detection of the pre-neoplastic lung lesions. Thus, the autoantibody chip conclusively out-performed WLB in the validation cohort. With respect to AFB, the autoantibody chip was not quite as sensitive (but comparable) and had a higher specificity. The specificities of the AAH and SCD classifiers were lower than expected (based upon expectations of application of the technique for the detection of NSCLC). One possibility might be due to the presence of AAH or SCD lesions in the control population used for the validation study. If some control subjects harbored these lesions, then this would reduce the specificity of the biomarker panel used in the array. In order to increase the specificity of the classifiers, control subjects could be screened for pre-neoplastic lesions to confirm their "lesion free" status.

### Tumor auto-antibody array proteins

The lesion specific proteins utilized in the array are shown in Table 3. For AAH lesions, 9 proteins gave the best sensitivity and specificity for the array. Following sequencing and identification, 7 of the 9 proteins have known malignant association from literature reports. Dysregulation of Latent Growth Factor beta Binding Protein (LTBP1) has been identified in asbestos-related lung tumors and was associated with DNA copy number alterations and tumor-associated miRNAs [16]. Furthermore LTBP1 may play a key role in the pathogenesis of mesothelioma via regulation of TGFβ activation in tumor tissue [17]. In healthy individuals, the expression of GAGE occurs in germ cells only [18]. However, GAGE proteins are expressed in a wide range of cancers, including stomach cancer, esophageal carcinoma and neuroblastoma, indicating a role in tumorigenesis [19]. GAGE7 is known for its anti-apoptotic action in human tumor cells; by conferring resistance to FasL-mediated apoptosis [20]. BMI1 expression has been associated with tumor invasion and metastasis in lung and breast carcinoma [21,22]. In malignant lung tissue, levels of the cell adherence molecule E-cadherin were inversely correlated with BMI1 [21]. Furthermore, *in vitro* studies showed that cigarette smoke condensate exposure significantly repressed miR-487b in normal respiratory epithelial cells and lung cancer cell lines. Subsequent experiments demonstrated that miR-487b directly targeted SUZ12, BMI1, WNT5A, MYC, and KRAS. Repression of miR-487b correlated with overexpression of these targets in primary lung cancers [23]. HES1 is a downstream effector protein of the NOTCH pathway. Westhoff *et al.*[24] reported that the NOTCH pathway has been shown to be altered in approximately one third of NSCLCs and that activation of the NOTCH pathway is associated with cell survival and poor clinical outcome. Interestingly, microarray analysis of human bronchial epithelial cells from smokers and smokers with COPD demonstrated that 45 of 55 Notch-related genes are expressed in the small airway epithelium, and that down-regulation of NOTCH pathway gene expression was associated with smoking and COPD [25]. Phosphodiesterase 4A (PDE4A) has been implicated in epithelial mesenchymal transition (EMT) of lung epithelial cells. Upregulation of this enzyme occurred in response to induction of EMT by treatment of A549 lung epithelial cells with TGFβ [26]. Furthermore, increased PDE4A expression and activity was associated with cell proliferation and angiogenesis [27]. NEFH [28] and HSPA8 [29,30] were also associated with human tumorigenesis, however there are no reports suggesting AGBL5 (an ATP/GTP binding protein) has malignant association outside of the current study.

With respect to SCD, a combination of 13 proteins gave the best sensitivity and specificity in the array. LTBP1, GAGE7 and BMI1 were present in the SCD panel, highlighting that these proteins potentially share similar biology with AAH lesions. Neuropilin-1 (NRP1) is a co-receptor for vascular endothelial growth factor (VEGF), and hence is associated with angiogenesis and tumor growth. NRP1 expression was assessed in whole sections of 65 primary breast carcinomas, 95 primary colorectal adenocarcinomas, and 90 primary lung carcinomas. Immunoreactivity for NRP1 was seen in vessels from normal tissues adjacent to cancer and in 98-100% of carcinomas. Tumor cell expression of NRP1 was also observed in 36% of primary lung carcinomas and 6% of primary breast carcinomas, but no colorectal adenocarcinomas [31]. Due to the lowered overall survival rate in NSCLC patients who expressed high levels of NRP1 in tumors, NRP1 has been proposed as a potential drug target for treatment of NSCLC [32]. Thioredoxin-like 2 (TXNL2) is a redox protein that has been reported to regulate cell growth and metastasis in breast cancer cells, and increased expression was associated with lower patient survival rates [33]. As tumor cells are known to produce higher levels of reactive oxygen species (ROS), proteins like TXNL2 help the cell to counteract these ROS, and hence continue to survive [33]. Conversely, increased mRNA expression of TXNL2 was correlated with prolonged patient survival following surgery of colorectal cancer [34]. Kallikrein-2 (KLK2) is a member of the Kallikrein superfamily of serine proteases, which are considered putative biomarkers for the screening, diagnosis, prognosis, and monitoring of various cancers including those of the prostate, ovaries, breast, testicles, and lung [35]. The most well-known kallikrein is prostate-specific antigen (KLK3), which is used clinically to diagnose human prostate cancer. KLKs are expressed in many human tissues and are regulated by steroid hormones [36]. Peroxiredoxin 4 (PRDX4) is a redox protein located in the endoplasmic reticulum and is a proposed scavenger enzyme for H_2_O_2_[37]. PRDX4 has been proposed as a biomarker of oxidative stress and has been associated with high risk of cardiovascular disease (CVD) and CVD mortality [38]. Studies in endothelial cells of patients with NSCLC showed increased expression of PRDX4 in tumors, which was not the case in adjacent normal tissue. Furthermore, elevated expression of PRDX4 was present in the epithelial cells of the tumors [39]. Baculoviral IAP repeat containing 3 (BIRC3) is a member of the IAP family of proteins which inhibit apoptosis by binding to tumor necrosis factor receptor-associated factors TRAF1 and TRAF2 [40]. Due to the ability of these proteins to inhibit apoptosis via the NfkB pathway, IAPs have been proposed as potential target molecules for anti-cancer therapeutics [41,42]. Nuclear over-expression of IAP1 was strongly correlated with tumor stage/grade and poorer prognosis in bladder cancer patients [43]. Elevated expression of specific members of the IAP family may be tumor specific, as studies in breast cancer cells showed elevated expression of cIAP2, where other members of the IAP family (cIAP1 and XIAP) were not upregulated [44]. Endoplasmic reticulum protein retention receptor 1 (KDELR1) has been implicated in the initiation of pro-apoptotic endoplasmic reticulum stress responses [45]. Data in relation to its involvement with tumorigenesis is currently very thin. Studies by Yi *et al.*[46] have reported that NPAS2 (involved with regulation of circadian rhythm) has associations with risk of breast cancer, prostate cancer and non-Hodgkins lymphoma. *In vitro* studies in breast cancer cell line MCF-7 by the same group, showed that KDLER1 is a transcriptional target of NPAS2 [46], however no studies to date have demonstrated a direct malignant association. The Sec61 complex is the central component of the protein translocation apparatus of the endoplasmic reticulum (ER) membrane, and is involved in the translocation of proteins into the ER membrane, notably the EGF receptor [47]. SEC61β expression was elevated 1.9 fold in human colorectal tumors and was proposed as a biomarker for the early detection of colorectal cancer. Furthermore, serum auto-antibodies to SEC61β were proposed as a surrogate biomarker for tissue SEC61β achieving a sensitivity and specificity of 79% and 75% respectively [48]. Leucine-rich repeat containing G protein-coupled receptor 6 (LGR6) is a member of the rhodopsin-like seven transmembrane domain receptor superfamily and mutations in this gene have been reported in colon cancer tissue [49] and gastric carcinoma [50]. Mechanistic studies have demonstrated that LGR6 is a high affinity receptor for R-spondins 1–3 and potentially functions as a tumor suppressor [49]. Finally, with respect to FZD6TFIP11, there are no published data to date on this protein.

### Considerations for use of the array

The use of tobacco, especially cigarette smoking, has long been considered as the leading cause of small cell and non-small cell lung cancer, contributes to 80% and 90% of lung cancer deaths in women and men, respectively [51]. However, not all smokers will develop lung cancer; only about 15% of heavy smokers are diagnosed with lung cancer in their lifetimes [52]. Therefore, it is critical to identify these individuals who have potential to develop lung cancer from high-risk smoking populations. The autoantibody chip developed in this study will be a good approach in identifying the pre-neoplastic lung lesions from the high-risk populations. By combining with the current imaging and endoscopy techniques, affected individuals could be identified earlier and hence receive appropriate medical guidance and monitoring to reduce their risk of developing lung cancer.

Three key limitations of the auto-antibody array need to be considered prior to its use in population studies. The first of these is that the classifiers for AAH and SCD do overlap and furthermore, share similarities with diseased tissue (Tables 1, 2 & 3). The overlap is to be expected however, as the dysregulation observed at the pre-neoplastic level is likely to share common irregularities and also to be causative of the malignant disease, and hence present in malignant tissue [6]. In order to measure the progression/regression of pre-neoplastic lung lesions in human population studies with this technology, it would be necessary to confirm that study participants do not harbor malignant disease (all cancers) at screening. Once confirmed to be cancer-free, any subsequent incidence of lung cancer is likely to have come from pre-neoplastic lesions either present at screening, or which have developed over the course of the study. To exclusively monitor development of pre-neoplastic lesions, study subjects should also be screened for these lesions using AFB and examined by a thoracic surgeon upon study commencement.

Secondly, it was assumed that as the classifiers were developed from pre-neoplastic lung tissue, they are specific for lung tissue lesions. As discussed above, it is evident that the array proteins share common dysfunctional pathways with other malignancies and hence false positives could be detected following application of serum from subjects with other types of cancer. Therefore, further studies are recommended to investigate the specificity of the classifiers for other types of cancer.

Thirdly, the auto-antibody array detects the presence of pre-neoplastic/malignant lesions alone. So far, no studies have attempted to correlate the strength of an autoantibody signal with the number or severity of lesions. Further studies are required to investigate the correlation of signal strength with lesion number and severity and conclusively determine if lesions are pre-neoplastic/malignant or not.

Given the advantages the auto-antibody array has in terms of the non-invasiveness of the sample collection (human blood), the higher throughput (compared to AFB, WLB and spiral CT), little need for experienced clinicians to interpret the data coupled with comparable sensitivity to AFB and higher specificity than AFB, we propose that this autoantibody array could be a useful tool in population screening programs to identify high risk individuals and studies undertaken to compare the harm reduction potential of combustible and non-combustible tobacco products, and nicotine delivery devices. Furthermore, a combination approach utilizing this auto-antibody array in conjunction with imaging technology could help to overcome potential cross-reactivity with other types of tumor.

## Conclusions

Our results showed promising accuracies for detection of pre-neoplastic lung lesions. This sensitive serologic test could be used as a tool for early diagnosis or screening of lung cancer, which could also be used in concert with radiographic imaging and other diagnostic strategies to facilitate earlier clinical intervention and provide useful information for assessing lung cancer risk in human populations.

## Materials and methods

### Study population

Following informed consent, 1500 high-risk patients (age > 55, smoking ≥ 20 pack-year) were registered at Hebei University Affiliated Hospital between 2002 to 2007. Serum samples were collected from all patients before any procedures were performed. All the patients were then gone through autofluorescence bronchoscopy (AFB) and spiral computed tomography (SCT) scanning. Biopsies were taken from all abnormal and suspicious areas during the bronchoscopy. Suspicious nodules found by SCT were surgically removed. All tissue specimens either from biopsies or surgeries were evaluated by pathologists. Confirmed SCD or AAH cases were selected for our study. Three pieces of AAH and three pieces of SCD tissues were collected from six patients and submerged into RNAlater buffer (Qiagen) immediately for cDNA library construction. In addition, 600 serum samples from 150 patients with AAH, 150 patients with SCD and 300 risk-matched control individuals were obtained through protocols approved by the Institutional Review Board. Standard venipuncture technique was used to draw peripheral blood into 10-ml glass red top tubes, and samples were left to stand for 30 minutes. Sera were then separated by centrifugation, and an aliquot was taken and frozen at -80°C. Detailed demographic and histopathologic data for serum samples is listed in Table 4.

**Table 4:** Demographic and histopathologic data for serum samples

	**Control training**	**Disease training**	**Control validation**	**Disease validation**
	**set No (%)**	**set No (%)**	**set N (%)**	**set No (%)**
**Sample N =**	100	100	200	200
**Age range (years)**	55-80	57-86	55-80	56-87
**Gender**				
Male	78 (78%)	79 (79%)	179 (89.5%	180 (90%)
Female	12 (12%)	11 (11%)	21 (10.5%)	20 (10%)
**Smoking (> 20 py)**				
Active	33 (33%)	36 (36%)	69 (34.5%)	73 (36.5%)
Former	64 (64%)	62 (62%)	127 (63.5%)	124 (62%)
Never	3 (3%)	2 (2%)	4 (2%)	3 (1.5%)
**Histopathology**				
AAH	0	50	0	100
BAC/AAH	0	0	0	39/100 (39%)
SCD	0	50	0	100
CIS/SCD	0	0	0	42/100 (42%)

### RNA extraction and T7 phage display library construction

Two T7-phage cDNA libraries were constructed using tissues from AAH or SCD patients. Total RNA was extracted and purified by using the RNeasy Mini Kit (Qiagen). PolyA mRNA was isolated from total RNA by Oligotex Direct mRNA Mini Kit (Qiagen) and mRNA concentration was measured by mRNA Concentration Module (Invitrogen). Equal amounts of mRNA from either 3 AAH or 3 SCD tissue samples were pooled and reverse transcribed to cDNA with random primers, ligated with EcoRI/HindIII linkers, and cloned into T7 select 10-3b vector arms of phage using the T7Select system (Novagen) according to the manufacturers instructions. After *in vitro* packaging, a phage library was obtained, and the titer of the phage library was tested using a plaque assay to determine the number of recombinants (inserts) generated within the library.

### Biopanning enrichment of disease associated phage clones

In order to enhance the selection of tumor-associated proteins, the AAH and SCD pooled phage library was biopanned using pooled AAH/SCD and normal serum samples. To remove non-tumor proteins, the library was affinity selected against antibodies in 6 pooled normal serum (250 μl pooled normal serum 1:20 dilution; 4°C o/n) bound to G agarose beads. Unbound phages were separated from bound phages by centrifugation. Phages expressing immunogenic tumor-associated proteins were selected with antibodies in serum pooled from 3 AAH and 3 SCD patients similarly bound to G agarose beads, and separated from unbound phages by centrifugation. The bound/reactive phages were eluted with 1% SDS and centrifugation. The eluted phage library thus expressed tumor proteins that had higher reactivity with antibodies found in AAH/SCD serum than those found in normal serum. This process was repeated four times with amplification in bacteria (E. coli BLT5615) between each biopan. The phages from biopan 4 were used to infect bacteria and grown on agar plates in limiting dilutions to determine titer and allow isolation of individual phage clones. Individual "plaques" on the agar plate, representing growth from a single phage clone, were usually seen at dilutions of 10^7^ to 10^8^. Phage colonies plated to limiting dilution were transferred to nitrocellulose membranes by plaque lift to be evaluated for reactivity with serum antibodies using standard immunodetection.

To confirm the enrichment of the biopanning, the outputs of biopans 1–4 (BP1-BP4) were plated onto LB-Agar plates (in limiting dilution) and plaque lifts were performed. The plaque lift nitrocellulose membranes were then incubated with pooled AAH/SCD patient serum (1:2000) followed by anti-human HRP-conjugated secondary Ab (1:1000) and detected with ECL chemiluminescence (Amersham). Immunodetection of each of the four biopans revealed an increasing number of reactive phage clones from BP1 to 4, and illustrates the ability of the sequential biopans to enrich the concentration of tumor-associated proteins recognized by antibodies in patient serum. To confirm the specific selection of disease-associated proteins, biopan 4 was plated onto LB-Agar plates and plaque lifts were performed twice with nylon disk membranes. One of the plaque lift membranes was then incubated with pooled AHH/SCD patient serum (1:2000) and the other was incubated with pooled normal sera, followed by anti-human HRP-conjugated secondary Ab (1:1000) and detected with ECL chemiluminescence (Amersham).

### Microarray high-throughput screening

After four cycles of biopanning, 4,000 individual phage colonies were picked from the biopanned library and inoculated into 96-well plates containing 150 μl E. coli BLT5615 cells in each well at 37°C for 3 hrs. After the incubation, 30 μl supernatant from each well was transferred into 384-well plates. "Empty T7" phage (T7 phage without inserts) clones were also incorporated into these 384-well plates as an internal control. OmniGrid 100 (Genomic Solution) microarray spotting machine was used to spot 5 nl phage lysates from each well of the 384-well plates onto nitrocellulose-coated membrane slides (Schott, Germany). In order to track the sample locations of each spot, a GenePix Array List (GAL) file was generated during spotting.

Five individual AAH or 5 SCD patient serum samples that were not used in the biopanning were used to identify possible tumor-associated proteins from the screening slides. Rabbit anti-T7 primary antibody was used to detect T7 capsid proteins as internal control for total proteins. Both preabsorbed plasma (serum:bacterial lysate, 1:30) samples and anti-T7 antibodies were diluted 1:3,000 with 4% dry-milk in TBST (1 ×TBS plus 0.1% Tween 20) and incubated with the screening slides for 1 hr at room temperature. Slides were washed and then probed with Cy5-labeled anti-human and Cy3-labeled anti-rabbit secondary antibodies; each antibody diluted 1:4,000 in 1× TBST together for 1 h at room temperature. Slides were washed again and then scanned with a GenePix 400B scanner.

Images were analyzed with GenePix 5.0 software (Axon Instruments/Molecular Devices, Union City, CA). Linear regression of the Cy5:Cy3 signals were generated using the same software. Phage clones that had Cy5:Cy3 signal ratios greater than 2 standard deviations from a linear regression were selected as candidates for use in the protein array.

### Testing serum samples using diagnostic chips

Four hundred immune-reactive phages identified by high-throughput screening (described above), plus 200 "empty" T7 phages, were combined, reamplified, and spotted in duplicate onto FAST slides as diagnostic chips. Replicate chips were used to assess 50 AAH or 50 SCD along with 100 control serum samples as a training group, according to the protocol described above for screening. The classifiers developed in the training group were then further validated with an independent blinded sample cohort as a validation group. A cohort of 400 serum samples consisting of 200 controls, 100 AAH and 100 SCD serum samples were tested using the same "diagnostic chips", and each sample’s statues was calculated separately using the AAH or SCD classifiers. The final results were checked with the true statuses of the samples, and the sensitivities and specificities were then calculated.

### Data processing and statistical model construction

The median Cy5 signal was normalized to the median Cy3 signal (Cy5:Cy3 signal ratio) as the measurement of human antibody against a unique phage-expressed protein. To compensate for chip-to-chip variability, measurements were further normalized by subtracting background reactivity of plasma against empty T7 phage proteins and dividing by the median of the T7 signal [(CY5:Cy3 of phage – Cy5:Cy3 of T7)/Cy5:Cy3 of T7]. Normalized Cy5:Cy3 ratio for each phage clone was independently analyzed for statistical significance between the patient (50 AAH or 50 SCD) samples and 100 control serum samples by *t*-test, using JMP statistical software (SAS, Inc., Cary, NC). Candidate phage markers were chosen if *P* < 0.01, and subjected in different combinations for classifier development. In order to develop statistical models/classifiers that generate the maximal accuracy for prediction, logistic regression (LR) was used for developing a model for disease prediction.

The calculation was carried out using SAS statistical software. Weights for the relative importance of each protein were developed and incorporated into the model. ROC curves based on logistic regression or an alternative method were used to determine an optimal (high sensitivity and specificity) decision rule for disease diagnosis. The classifiers were further examined by leave-one-out cross-validation.

### Sequence identification

Identities of the phage cDNA inserts used in the classifiers were identified through PCR-amplification using commercially available T7 phage vector primer (Novagen, USA). The sequences are: T7 up 5′ - GGAGCTGTCGTATTCCAGTC - 3′ and T7 down 5′ - AACCCCTCAAGACCCGTTTA - 3′. The PCR products were purified, and sequenced. The sequence results were identified in the GenBank database using the BLAST search program.

## Competing interests

The authors declare that they have no competing interests.

## Authors’ contributions

FJL oversaw this study, and participated in the experimental design and manuscript writing. WS carried out data mining and statistical analysis. JZ participated in clinical specimen collection and staging. JL and HW participated in phage-display and protein microarray study. XZ participated in the sequence alignment and drafted the manuscript. LZ conceived the study, and participated in its design and coordination and helped to draft the manuscript. All authors read and approved the final manuscript.
